# ADInteractome: discovering Alzheimer’s disease-associated gene interactions from single-nucleus RNA-seq data through neural interaction states

**DOI:** 10.1093/bib/bbag601

**Published:** 2026-11-02

**Authors:** Yifan Wu, Neha Maurya, Yuntao Yang, Wenbo Chen, Guanchu Wang, Zirui Liu, Xia Hu, W Jim Zheng, Zhaozhuo Xu

**Affiliations:** College of Science and Engineering, University of Minnesota Twin Cities, Mayo D528, 420 Delaware St SE, Minneapolis, MN 55455, United States; McWilliams School of Biomedical Informatics, UTHealth Houston, 7000 Fannin St, Suite 600, Houston, TX 77030, United States; MD Anderson Cancer Center, 1515 Holcombe Blvd, Houston, TX 77030, United States; McWilliams School of Biomedical Informatics, UTHealth Houston, 7000 Fannin St, Suite 600, Houston, TX 77030, United States; Department of Computer Science, University of North Carolina at Charlotte, Woodward Hall, 9201 University City Blvd, Charlotte, NC 28223, United States; Department of Computer Science & Engineering, University of Minnesota Twin Cities, 4-192 Keller Hall, 200 Union Street SE, Minneapolis, MN 55455, United States; Department of Computer Science, Rice University, Duncan Hall 3122, 6100 Main Street, Houston, TX 77005, United States; McWilliams School of Biomedical Informatics, UTHealth Houston, 7000 Fannin St, Suite 600, Houston, TX 77030, United States; School of Computing, Stevens Institute of Technology, Gateway Center North, 1 Castle Point Terrace, Hoboken, NJ 07030, United States

**Keywords:** gene interactions, Alzheimer’s disease, neural interaction states

## Abstract

Alzheimer’s disease (AD) is a prevalent neurodegenerative disorder characterized by amyloid *β * plaques and tau tangles, yet its molecular mechanisms remain elusive. Advances in single-nucleus RNA sequencing (snRNA-seq) have provided profound insights into the cellular heterogeneity of AD-associated brains. In this study, we introduce ADInteractome, an interpretable artificial intelligence framework designed to discover gene interactions associated with AD directly from the internal state of neural networks trained on the snRNA-seq data of brain cells. The method is based on the intuition that if a neural network can accurately classify cells from AD and non-AD donors based on their snRNA-seq data, then its internal interaction states, reflecting interaction patterns within the gene expression data, are informative for identifying biologically meaningful gene interactions. Applied to a large-scale snRNA-seq dataset from the Seattle Alzheimer’s Disease Brain Cell Atlas, ADInteractome reveals cell-type-specific and AD-associated gene circuits supported by existing literature. The results were further supported by our virtual knock-out and knock-in experiments, a novel concept to probe our findings through a data-driven approach. Compared with traditional co-expression network methods, it achieves stronger enrichment for known AD-associated gene interactions and uncovers novel interaction patterns. These findings highlight the potential of interpretable AI approaches for elucidating complex molecular interactions in neurodegenerative disease.

## Introduction

Alzheimer’s disease (AD) [1] is a progressive neurodegenerative disorder and the leading cause of dementia among older adults, affecting an estimated 5.8 million Americans [2]. Despite extensive studies of hallmark features such as amyloid *β * (A*β *) plaques and tau tangles [3, 4], the molecular mechanisms underlying AD remain poorly understood, and no effective therapeutic strategy exists to cure or alter its progression [5]. Advances in single-cell transcriptomics, particularly single-nucleus RNA sequencing (snRNA-seq), have enabled transcriptomic profiling at cellular resolution, revealing cell-type-specific alterations across diverse neural and glial populations [6, 7]. Large-scale resources such as the Seattle Alzheimer’s Disease Brain Cell Atlas (SEA-AD) [8] provide unprecedented opportunities to analyze AD at cellular and molecular levels by combining rigorous neuropathological annotations with gene expression profiles across more than one million nuclei.

While these datasets are rich, most computational approaches for inferring gene interactions rely on predefined biological knowledge, such as protein–protein interaction networks, gene regulatory maps, or transcription factor binding profiles [9–16]. Although such priors can improve interpretability, they also introduce biases that constrain discovery and may hinder the identification of novel or context-specific interactions [17, 18]. Previous studies have noted that many computational models enforce strong structural assumptions based on these priors, limiting their flexibility and capacity to capture hidden biology [19–21]. We instead advocate a purely data-driven approach that allows snRNA-seq data itself to define the interaction landscape, enabling unbiased discovery of molecular relationships in AD.

Recent advances in artificial intelligence (AI), particularly Transformer models [22], have achieved strong performance in single-cell analysis, with several architectures developed specifically for scRNA-seq and snRNA-seq data [23, 24]. These models excel at cell-type classification but remain underexplored for inferring biologically meaningful gene interactions. The self-attention mechanism in Transformers is explicitly designed to capture feature dependencies [22], making it a promising foundation for interaction discovery. However, extracting robust signals from attention maps is challenging, and conventional implementations are computationally demanding, limiting their scalability to datasets of millions of nuclei.

Here, we introduce **ADInteractome**, a scalable and interpretable AI framework that discovers AD-associated gene interactions directly from snRNA-seq data. The methodological innovation lies in treating the *internal states of neural networks* as a source of biological meaningful information. *If a neural network can accurately classify AD-associated cells, then its hidden representations must encode informative patterns of gene interactions.* We formalize these patterns as *neural interaction states* and design scalable algorithms to aggregate them across layers, heads, and cells. This enables us to rank gene pairs by their contribution to AD classification and construct cell-type-specific interaction graphs.

Unlike conventional attention analyses that are resource-intensive and often opaque, ADInteractome provides a lightweight and scalable mechanism to extract robust gene interaction signals. Applied to SEA-AD, it identifies hub genes with emerging genetic links to AD, such as *DLG2* [25], together with underexplored partners, generating mechanistic hypotheses for cell-type-specific circuits disrupted in AD. By leveraging internal neural states, ADInteractome demonstrates how modern AI can move beyond prediction to *explanation*, offering a scalable paradigm compared with existing solutions for uncovering gene interactions from large-scale single-cell data.

## Materials and methods

The overall workflow and results of ADInteractome are summarized in Fig. 1. Briefly, snRNA-seq gene expression profiles from SEA-AD were first used as model inputs to train neural networks for AD-association classification. We then extracted neural interaction states from the trained models, including attention-based interaction signals from CelluFormer and weight-derived interaction signals from Multilayer Perceptron (MLP) models. These interaction states were aggregated to rank AD-associated gene–gene interactions, followed by downstream enrichment analysis, cell-type-specific interaction graph construction, and hub-gene analysis. The following sections describe each component of this workflow in detail.

**Figure 1:**
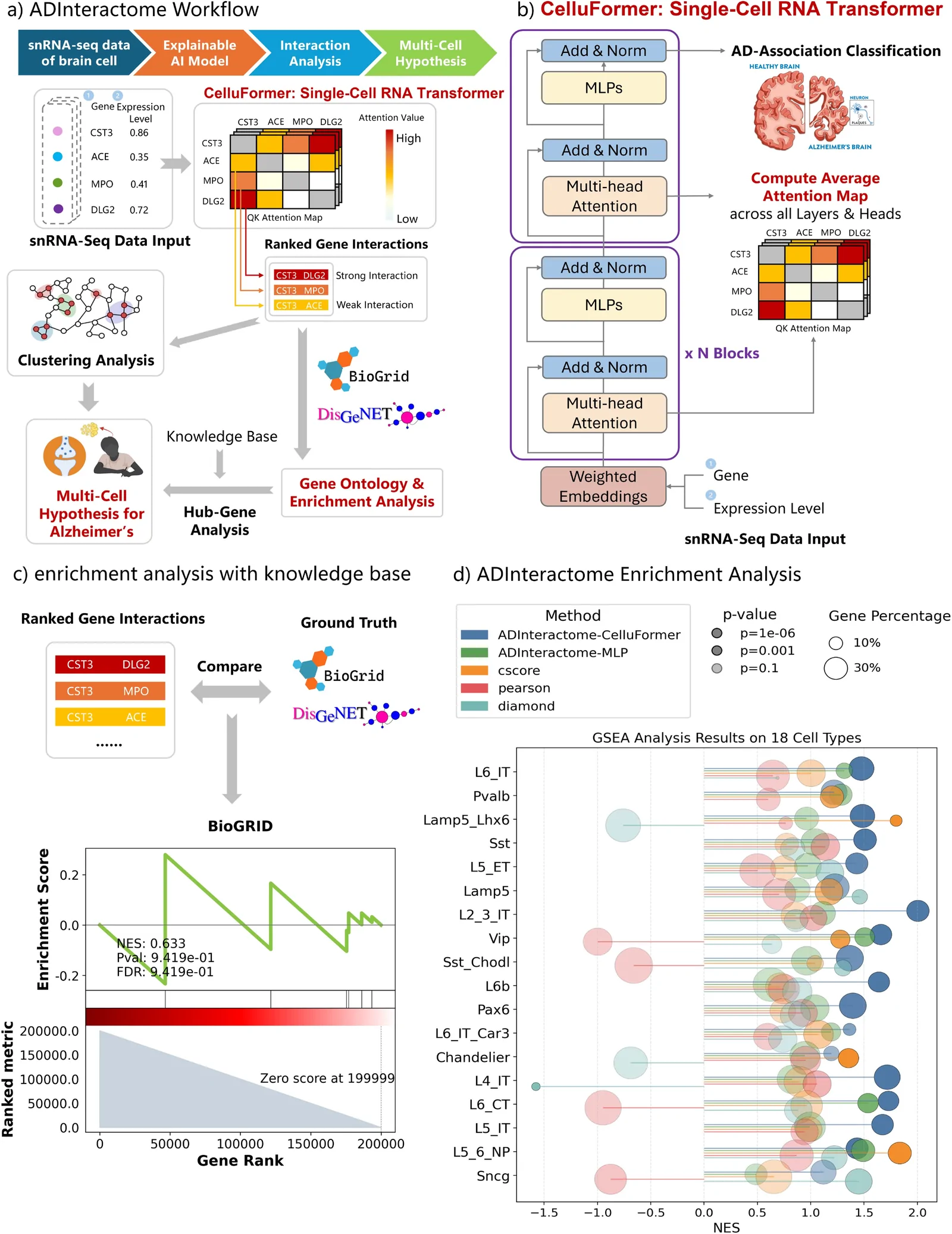
Workflow of the ADInteractome framework. (a) Overall workflow for identifying AD-associated gene–gene interactions from snRNA-seq data. (b) Single-cell RNA Transformer architecture (CelluFormer) for AD-association classification and attention-based interaction extraction. (c) Enrichment analysis comparing ranked gene interactions with BioGRID and DisGeNET knowledge bases. (d) Comparative enrichment results across 18 neuronal cell types and baseline methods.

### Data preprocessing

We utilized the SEA-AD dataset [8], which includes snRNA sequencing data of 36 601 genes from 84 senior brain donors characterized by varying levels of AD neuropathological changes, ranging from high to no AD. SEA-AD has supported multiple AI-related research [26, 27]. For a comparative analysis between AD-associated and normal brains, we selected 42 donors from the high-AD category and 9 from the non-AD category, based on their neuropathological classifications. The dataset provides annotations across 24 cell types. To train the neural network model, we select 18 cell types, as shown in Table 1, for our study.

**Table 1:** Weighted F1 scores of CelluFormer Transformer and MLP classifiers across cell types. The MLP classifier was also used as a neural-network comparison model for interaction ranking.

Cell type	CelluFormer_f1	MLP_f1
L4_IT	0.9908	0.9826
L5_6_NP	0.9896	0.9777
Lamp5	0.9530	0.9533
L6_IT	0.9959	0.9853
Vip	0.9668	0.9555
Pax6	0.9804	0.9469
Chandelier	0.9551	0.9474
Sst_Chodl	0.9283	0.9554
L2_3_IT	0.9922	0.9804
L5_ET	0.9962	0.9615
Sncg	0.9568	0.9363
L6_CT	0.9906	0.9653
L5_IT	0.9941	0.9816
L6_IT_Car3	0.9921	0.9711
Lamp5_Lhx6	0.9708	0.9730
Sst	0.9543	0.9385
Pvalb	0.9779	0.9560
L6b	0.9890	0.9711

The binary AD-association label used in this study is derived from donor-level SEA-AD neuropathological classification rather than from cell-level expression patterns or model-derived criteria. Cells from donors annotated as High Alzheimer’s disease neuropathologic change (High ADNC) were assigned the AD-associated label, and cells from donors annotated as No AD were assigned the non-AD label. Low and Intermediate ADNC donors were excluded from the binary classification analysis because these donors represent mixed or transitional neuropathological states that would introduce ambiguity into a strict AD versus non-AD formulation. Thus, every nucleus inherits the label of its corresponding donor.

### ADInteractome: gene interactions discovery from neural interaction states

In this work, we introduce ADInteractome, a unified framework for discovering AD-associated gene interactions from snRNA-seq data. ADInteractome consists of three key steps: (i) training neural networks to classify cells as AD-associated or not, (ii) extracting internal interaction states from the trained models, and (iii) constructing gene–gene interaction graphs to reveal cell-type-specific molecular circuits. This framework enables systematic analysis of gene–gene interactions and the generation of biologically testable hypotheses across diverse neural cell types.

#### Formulating neural interaction states from neural networks

snRNA-seq data for a cell can be formulated as a set of gene expression profiles. We denote the snRNA-seq dataset as * X *, where each cell * x ∈ X * is represented as a set of gene-expression pairs. Specifically, a cell * x * with * k * expressed genes can be represented as: $ x = \{(v_{1}, u_{1}), (v_{2}, u_{2}), \ldots , (v_{k}, u_{k})\} $ where each tuple * (v, u) * indicates that gene * v ∈ [V] * is expressed with an expression level $ u \in \mathbb{R} $. Here, * V * denotes the total number of genes observed across the dataset * X *. This formulation highlights that snRNA-seq data for a cell is inherently a set of gene expression measurements, with different cells expressing different subsets of genes. Even when two cells express the same gene, their expression levels may vary. According to the SEA-AD dataset, each cell * x ∈ X * has a binary label $ y \in \{0,1\} $ indicating its association with AD. A label of * 1 * denotes that the cell is AD-associated.

ADInteractome aims to identify significant gene–gene interactions within the vocabulary * V * that contribute to complex biological processes and disease mechanisms associated with AD. This approach is based on the premise that if a neural network can accurately classify cells as AD-associated or not using their snRNA-seq data, then the neural network’s internal states, which capture interaction patterns within the gene expression data, can reveal biologically meaningful gene–gene relationships associated with AD. To implement this, ADInteractome first trains neural networks to perform binary classification, predicting whether a cell is AD-associated based on its snRNA-seq data * x *. We denote this neural network as a function * f *, which outputs the AD-association prediction for each cell * x *. Since each cell * x * is represented as a set of gene expressions, * f * is equipped with an embedding table $ W^{0} \in \mathbb{R}^{V \times d} $, where each row corresponds to a * d *-dimensional representation vector for a gene. Given the input $x = \{(v_{1}, u_{1}), (v_{2}, u_{2}), \ldots , (v_{k}, u_{k})\}$, * f * retrieves the representation vector $ W^{0}_{v} $ for each expressed gene * v * and scales it by the corresponding expression level * u *. The neural network *f* then applies a series of input-weight multiplications to these genes’ internal states, progressively transforming them into a binary prediction indicating whether the cell is AD-associated.

Once the function *f* is trained on *X* with binary AD-association labels *y* assigned to each cell *x∈ X* and achieve decent generalization performance in classifying the AD-association from snRNA data of cells other than *X*, we can extract internal states of two genes $v_{i}, v_{j}$ from the computing workflow of *f* that suggest their interaction. Formally, we define these internal states as neural interaction states. In this work, we provide the formulation of neural interaction states for different architectures of neural network *f*, from simple fully-connected networks to advanced Transformer models [22].

If a neural network *f* is instantiated with *L* fully-connected layers, we formulate the neural interaction states between gene $v_{i}$ and $v_{j}$ as:


(1) \begin{align*} s_{i,j} = \langle{\mathbf{1}^{\top}|W^{L}|\,|W^{L-1}|\cdots|W^{1}|}, \min(|W^{0}_{i}|, |W^{0}_{j}|) \rangle.\end{align*}

Here, $W^{l}\in \mathbb{R}^{d_{l}\times d_{l-1}}$ denotes the weight matrix of the *l*th fully-connected layer, with $d_{0}=d$ and $d_{L}=2$ output units corresponding to the Not AD and High AD classes, $\mathbf{1}\in \mathbb{R}^{d_{L}}$ is the all-ones vector that aggregates over the output units, *|· |* denotes the element-wise absolute value, and $\min (\cdot ,\cdot )$ is taken element-wise. This formulation measures the inner product between two components: (i) the cumulative impact of *f*’s weights’ magnitudes towards the binary prediction of AD-association, (ii) the element-wise minimum magnitudes of gene representations vector in the embedding table for genes $v_{i}$ and $v_{j}$. The computing workflow of a fully-connected network ensures the dimensionality consistency of the two components. Empirical evidence suggests that this measure [28] is an effective indicator for extracting feature interactions suggested by fully-connected networks.

We view the self-attention mechanism in Transformers applied to a cell’s set-style snRNA gene expressions as a pathway to extract neural interaction states. Let *f* be a Transformer model with *L* self-attention layers, each containing *H* heads. For an input cell *x* with *k* expressed genes, each head in each layer produces an attention map $A^{l,h}(x) \in \mathbb{R}^{k \times k}$. We define the *(i,j)*th entry of $A^{l,h}(x)$ for layer *l ∈ [L]* and head *h ∈ [H]* as the neural interaction state between gene $v_{i},v_{j}$ expressed in input cell *x*:


(2) \begin{align*} s^{{l,h}}_{i,j}(x) = A^{l,h}_{i,j}(x).\end{align*}

For notational simplicity, the subscripts *i* and *j* of $A^{l,h}(x)$ refer to the positions of genes $v_{i}$ and $v_{j}$ in the input set of cell *x*. This formulation captures the intuition that the Transformer explicitly models gene–gene interactions through its attention mechanism. We note that the neural interaction states in Transformer models are specific to each cell. To identify gene–gene interactions at the dataset level, these cell-specific interaction states must be aggregated across many cells. Importantly, we do not interpret a single raw attention weight as direct evidence of a mechanistic molecular interaction. Attention values can be affected by token competition, sparsity, expression prevalence, scaling, and the training objective. In ADInteractome, attention-derived states are therefore used as model-based ranking signals for hypothesis generation, and the resulting ranked interactions are evaluated using external biological evidence, enrichment analysis, network structure, and virtual perturbation experiments.

#### Ranking gene–gene interactions with scores derived from neural interaction states

Once the neural interaction states are obtained from neural networks trained to classify a cell’s AD association using its snRNA profile, we use these states to compute a ***neural interaction score*** that ranks gene–gene interactions by their association with AD. In this study, we employ two types of architectures to instantiate the neural network *f*, i.e. fully-connected networks and the Transformer models. Accordingly, we describe the computation of the neural interaction score from the neural interaction state of both models below.

For fully-connected networks, the neural interaction state for a gene pair, as defined in Equation (1), is computed directly from the weights of the trained network. Once the neural network *f* is trained on the dataset *X* with AD-association labels, these weights can be used to derive an interaction score for each gene pair. Since Equation (1) ensures that $s_{i,j}$ is always non-negative, we can directly use $s_{i,j}$ as the neural interaction score to rank gene–gene interactions by their association with AD.

For Transformer models, neural interaction states between gene $v_{i},v_{j}$ expressed in cell *x*, as suggested by Equation (2), are computed across diverse layers and heads in the Transformer model. To compute a single, non-negative neural interaction score for ranking the AD-association of gene–gene pairs, we perform the following accumulation across different layers and cells in *X*. Let $\mathcal{X}_{ij}=\{x\in X: v_{i} \ \mathrm{and} \ v_{j}\ \text{are jointly observed in}\ x\}$ denote the set of cells in which the two genes are co-observed. The Transformer-based neural interaction score is computed as


(3) \begin{align*} S_{i,j} = \frac{1}{LH|\mathcal{X}_{ij}|} \sum_{x\in \mathcal{X}_{ij}} \sum_{l\in [L]} \sum_{h\in[H]} {\sigma\bigl(Z^{l,h}(x);T\bigr)_{i,j}},\end{align*}

where $Z^{l,h}(x)=Q^{l,h}(x)K^{l,h}(x)^{\top }/\sqrt{d_{k}}\in \mathbb{R}^{k\times k}$ denotes the presoftmax attention logits of head *h* in layer *l*, with $d_{k}$ the per-head key dimension, so that $A^{l,h}(x)=\sigma (Z^{l,h}(x);1)$, and $\sigma (Z;T)_{i,j}=\exp (Z_{i,j}/T)/\sum _{j^{\prime}}\exp (Z_{i,j^{\prime}}/T)$ is the row-wise temperature softmax [29, 30]. This co-occurrence-normalized formulation estimates the mean neural interaction signal conditional on both genes being observed, rather than inflating scores for gene pairs that simply occur in more cells. We use *T=3.0* as the default setting and report temperature-sensitivity analysis in Supplementary Table S1.

#### Enrichment analysis of ranked gene–gene interactions

For a comprehensive evaluation of the ADInteractome-ranked gene–gene interactions, we employed the Kolmogorov–Smirnov test [31] using the GSEApy package [32] in Python. We used the normalized enrichment score (NES) [33] as the evaluation metric. The a priori gene–gene pair set was constructed from *BioGRID* [34] and *DisGeNET* [35]. We first extracted genes associated with AD from DisGeNET, then identified gene–gene pairs in BioGRID in which at least one gene appeared in this AD-associated gene set. This process yielded a filtered BioGRID dataset containing gene–gene pairs relevant to AD. We then computed the NES for the ADInteractome-ranked gene–gene pair list against this filtered set to evaluate whether ADInteractome provides stronger enrichment for known AD-associated gene–gene interactions. We also compared ADInteractome with alternative ranking methods, allowing us to assess its relative strength in prioritizing biologically meaningful gene interactions.

ADInteractome produces a ranked list of gene–gene interactions for each cell type by extracting neural interaction states from trained neural networks. These rankings naturally form a graph for each cell type, denoted as the cell-type-specific gene interaction graph, providing a clear visualization of the gene interactions identified by ADInteractome. Using the top 1000 ranked gene–gene pairs produced by ADInteractome from neural networks trained on the SEA-AD snRNA-Seq dataset, we form gene interaction graphs for the cell types Chandelier, L6 CT, L6 IT Car3, L5 ET, and L5/6 NP. The number of unique genes contributing to these networks for each cell type was as follows: Chandelier (756), L6 CT (775), L6 IT Car3 (843), L5 ET (854), and L5/6 NP (501). From these, the top gene clusters identified through k-means clustering in STRING (*k=3* for all cell types; the largest cluster was retained) consisted of 535 genes for Chandelier, 580 for L6 CT, 643 for L6 IT Car3, 659 for L5 ET, and 327 for L5/6 NP. Next, we perform gene set enrichment analysis [21] using DISEASES database [36] to show how the gene clusters obtained from ADInteractome-produced the cell-type-specific gene interaction graphs enrich our knowledge regarding specific brain-related diseases. We also perform reactome pathway enrichment analysis [37] for the top gene clusters obtained by clustering on cell-type-specific gene interaction graphs.

This quantitative enrichment analysis illustrates how ADInteractome develop upon existing knowledge related to AD. It helps bridge the final gap in analyzing discovered gene interactions using neural interaction states.

### Virtual knock-out knock-in experiment for evaluating the hypothesis generated by ADInteractome

To validate the top-ranked gene–gene interactions identified by ADInteractome in support of the multi-cell-type hypothesis, we perform a virtual knock-out knock-in experiment focusing on DLG2-centered pairs. The experiment tests whether introducing a gene–gene pair $(v_{i}, v_{j})$ into cells within the snRNA-seq dataset *X* leads to a measurable improvement in the Celluformer model’s accuracy in predicting AD association. A significant increase in predictive performance after knock-out and knock-in suggests that the gene pair may be associated with AD.

Here, “knock-in” refers to a masking-based in silico perturbation experiment. Let $\mathbf{x}^{(0)}$ denotes the masked baseline input and let $P=\{v_{i},v_{j}\}$ denotes a selected gene pair. We define $\mathbf{x}^{(0)}\oplus P$ as the perturbed input in which the original expression values of genes in *P* are restored on top of the same masked baseline. Here, $\mathbf{x}^{(0)}$ corresponds to the masked cell $x_{\textsf{base}}$ defined below, $\mathcal{D}_{\mathbf{x}^{(0)}}$ denotes the masked dataset $X_{\textsf{base}}$ with the original AD-association labels, and $\mathcal{D}_{\mathbf{x}^{(0)}\oplus P}$ denotes the same dataset after restoring *P* in every cell. The accuracy change for pair *P* is


(4) \begin{align*} \Delta \mathrm{Acc}(P) = \mathrm{Acc}\!\left(f;\mathcal{D}_{\mathbf{x}^{(0)} \oplus P}\right) - \mathrm{Acc}\!\left(f;\mathcal{D}_{\mathbf{x}^{(0)}}\right).\end{align*}

A positive $\Delta \mathrm{Acc}(P)$ indicates that restoring the selected pair recovers predictive information under the same masking protocol. To reduce circularity concerns, we also apply the identical knock-in procedure to randomly sampled gene pairs and compare the resulting $\Delta \mathrm{Acc}$ distributions using a one-sided Mann–Whitney U test.

In this virtual knock-out knock-in experiment, as outlined previously, we consider a Celluformer model * f * with * L * self-attention layers and * H * attention heads, trained on the snRNA-seq dataset * X * to perform binary classification for AD association. Let * V * denote the set of genes expressed in at least one cell in * X *. For each gene pair $ v_{i}, v_{j} \in V $, ADInteractome provides the neural interaction states $ A_{i,j}^{l,h} $ at layer * l * and head * h * as shown in Equation (2).

The virtual knock-out knock-in workflow can be summarized as follows. We start by creating a base gene set $V_{\textsf{base}}\subset V$ such that for any cell *x∈ X*, there exists at least one gene $v\in V_{\textsf{base}}$ that is expressed. Next, we perform knocking out by masking the expression levels of all genes $v\in V\setminus V_{\textsf{base}}$ in cells from *X* as zero, resulting in a dataset $X_{\textsf{base}}$. Each cell $x_{\textsf{base}}\in X_{\textsf{base}}$ is a masked version of its original *x∈ X*. As expected, the predictive performance of *f* on $X_{\textsf{base}}$ for AD-association will significantly decline since many meaningful genes are masked. Finally, we virtually knock-in the interested gene pair $v_{i}, v_{j}$ to $X_{\textsf{base}}$, meaning that if *x∈ X* is expressed with $v_{i}$ or $v_{j}$, we will release the mask on them and retain their original expression value in *X* in the masked cell $x_{\textsf{base}} \in X_{\textsf{base}}$. Then, we observe the performance increase in *f* on the updated $X_{\textsf{base}}$ to evaluate how important the Celluformer thinks regarding gene pair $v_{i}, v_{j}$.

To construct $V_{\textsf{base}}$, we begin by ranking the genes in *V* using the neural interaction states from Celluformer. Specifically, for each gene $v_{i} \in V$ that is expressed in *X*, we compute


(5) \begin{align*} S_{i} = {\frac{1}{L H |\mathcal{X}_{i}|} \sum_{x \in \mathcal{X}_{i}}} \sum_{l \in [L]} \sum_{h \in [H]} {\sigma\bigl(Z^{l,h}(x); T\bigr)_{i,i}},\end{align*}

where $\mathcal{X}_{i}=\{x\in X: v_{i} \text{ is expressed in}\ x\}$ denotes the set of cells expressing $v_{i}$, and we extract the diagonal entries of each cell *x*’s attention map to measure self-importance. We then sort the genes in ascending order based on $S_{i}$. Finally, we select genes with the lowest $S_{i}$ values to form $V_{\textsf{base}}$, ensuring that each cell *x ∈ X* has at least one expressed gene included in $V_{\textsf{base}}$. Finally, we can use $V_{\textsf{base}}$ to create $X_{\textsf{base}}$ and then perform virtual knock-in for gene pairs suggested by the ADInteractome-generated hypothesis. The intuition behind this procedure is to perform a virtual knock-out, but include some basic, yet potentially AD-irrelevant genes, to ensure that every cell has some genes expressed. Next, we perform a virtual knock-in to see how ADInteractome supports gene–gene interactions identified by the multi-cell-type hypothesis.

## Results

In this section, we apply ADInteractome to SEA-AD snRNA-seq data for gene interaction discovery and provide a multi-cell-type hypothesis that DLG2 (Discs Large MAGUK Scaffold Protein 2) [25, 38, 39] forms a core hub across four cortical cell types: Chandelier cells [40], L5/6 NP (near-projecting) [41], L6 CT (Corticothalamic) [41], and L5 ET (Extratelencephalic) neurons [42], interacting with a set of conserved partners implicated in neurotransmission, calcium signaling, and synaptic remodeling. We also validate this hypothesis generated by ADInteractome with existing literature and a targeted virtual knock-in and knock-out experiment.

### Neural networks encode gene–gene interactions for high accuracy Alzheimer's disease-association classification of cells

Within the ADInteractome framework, we trained lightweight neural networks to classify cells as AD-associated or not across 18 neuronal cell types using snRNA-seq profiles from the SEA-AD dataset. These models included fully connected networks and Transformer-based architecture. We denote the Transformer model trained on SEA-AD as *Celluformer*. As shown in Table 1, all 18 models achieved a weighted F1 score >0.9, confirming high classification accuracy. This finding supports the central premise of ADInteractome: *that gene–gene interactions, when explicitly encoded as internal interaction states in neural networks, are sufficient to distinguish disease-associated from non-disease-associated cells*. The MLP classifier in Table 1 serves two purposes: it provides an architecture-level comparison for AD classification and supplies a non-attention neural baseline for extracting interaction scores from fully connected model weights.

### Neural interaction states reflect Alzheimer's disease-associated gene–gene interactions

ADInteractome defines neural interaction states as the gene–gene relationships encoded within trained neural networks. In fully-connected networks, denoted as MLP, we define the accumulated product of weights associated with two genes in the network as their interaction states. In CelluFormer, we explicitly use the attention scores between a pair of genes across layers as their neural interaction states. Next, we develop a neural interaction score to rank the gene–gene interaction suggested by the neural network for AD-association based on neural interaction states. To evaluate the ranked AD-associated gene–gene interactions suggested by ADInteractome, we employed the Kolmogorov–Smirnov test [31] using the GSEApy package [32] in Python. We used the NES [33] as the evaluation metric. The *a priori* gene–gene pair set was constructed from BioGRID[34] and DisGeNET [35]. We first extracted genes associated with AD from DisGeNET and then identified gene–gene pairs in BioGRID in which at least one gene was present in this AD-associated gene set. This process yielded a filtered BioGRID dataset containing gene–gene pairs relevant to AD. We then computed the NES for the ADInteractome-ranked gene–gene pair list against this filtered set to evaluate whether ADInteractome provides stronger enrichment for known AD-associated gene–gene interactions. We compared ADInteractome with alternative ranking methods, including Pearson correlation [43], CS-CORE [44], the MLP-derived neural interaction score, and a Diamond-inspired heuristic ranking proxy [45]. CS-CORE was included because it is designed for cell-type-specific co-expression inference from single-cell RNA-seq data, whereas the Diamond-inspired proxy was used only as a heuristic model-based baseline and should not be interpreted as a faithful implementation of the full false discovery rate (FDR) -controlled Diamond procedure. This comparison allowed us to evaluate its effectiveness in prioritizing biologically meaningful gene interactions. As shown in Fig. 1c, ADInteractome, when powered by Celluformer or MLP, achieves stronger enrichment for known AD-related gene relationships, with a Wilcoxon signed-rank *P*-value below 0.0001. Additional details on baseline configurations, including the limitations of the Diamond-inspired proxy, are provided in Supplementary Section S1.

### Gene interaction graphs reveal cell-type-specific molecular circuits

To explore the structure of the learned interactions, we constructed cell-type-specific gene interaction graphs as shown in Fig. 2 using the top 1000 ranked gene pairs from five neuronal cell types. k-means clustering (*k=3*) of these networks in STRING revealed distinct modules enriched for synaptic functions, including glutamate signaling, neurotransmitter receptor activity, calcium influx, and long-term potentiation. Although these pathways were broadly conserved, cell-type-specific enrichments emerged. For instance, L6 IT Car3 neurons were additionally enriched for Reactome terms related to insulin secretion and energy metabolism, and Chandelier cells for PLC-*β *-mediated events and muscle contraction; these terms are likely driven by shared calcium-signaling and ion-channel genes rather than by tissue-specific secretory or contractile processes. These results demonstrate that internal interaction states capture both shared and cell-type-specific regulatory programs relevant to AD. We used the top 1000 ranked pairs as the primary graph-construction threshold because this cutoff preserved sufficient connectivity for clustering while maintaining sparse, interpretable hub structure. Threshold-sensitivity analyses at the top 800 and top 1500 cutoffs are reported in Supplementary Table S5; the DLG2-centered signal was preserved at the top 800 cutoff but diluted when lower-confidence edges were added at the top 1500 cutoff.

**Figure 2:**
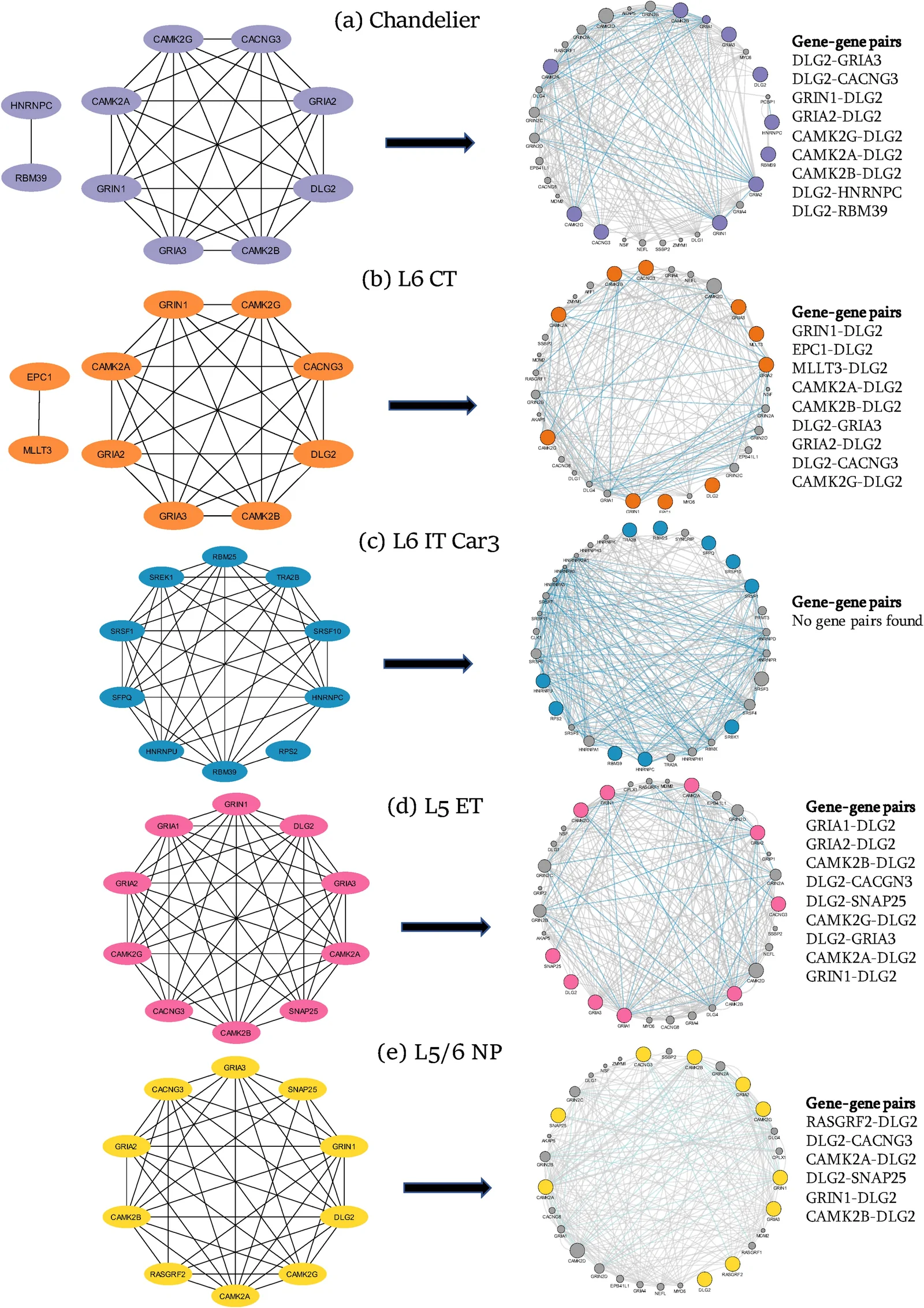
Gene–gene interaction graph with top interacting gene–gene pairs derived by neural interaction states.

Across four of the five neuron subtypes analyzed, *DLG2 consistently appeared as a central node in the top gene–gene interaction graphs*. DLG2, a postsynaptic scaffold protein, interacted with GRIA2, GRIA3, GRIN1, CAMK2A, CAMK2B, CAMK2G, SNAP25, and CACNG3, which are genes essential for synaptic signaling and plasticity. These patterns, derived from neural interaction states, highlight DLG2’s role as a molecular integrator of neurotransmission. In contrast, DLG2 was absent from the L6 IT Car3 network, suggesting cell-type-specific divergence. Notably, several DLG2 interactors, such as RBM39, EPC1, and MLLT3, are not currently established as AD-related but have emerged as top-ranked partners, pointing to underexplored regulatory mechanisms uncovered by the model.

### DLG2-centered multi-cell-type hypothesis generated with ADInteractome

As illustrated in Fig. 3, we propose a multi-cell-type hypothesis for AD centered on the gene DLG2, derived from ADInteractome analysis. DLG2 (Discs Large MAGUK Scaffold Protein 2) is a postsynaptic density protein in the MAGUK family, essential for organizing glutamatergic synapses. It scaffolds AMPA and NMDA receptor complexes and regulates intracellular signaling cascades crucial for synaptic plasticity [25, 39]. ADInteractome identifies DLG2 as a core hub gene across four cortical neuronal subtypes: Chandelier cells, L5/6 near-projecting (NP), L6 corticothalamic (CT), and L5 extratelencephalic (ET) neurons. In each, DLG2 interacts with conserved partners involved in neurotransmission, calcium signaling, and synaptic remodeling.

**Figure 3:**
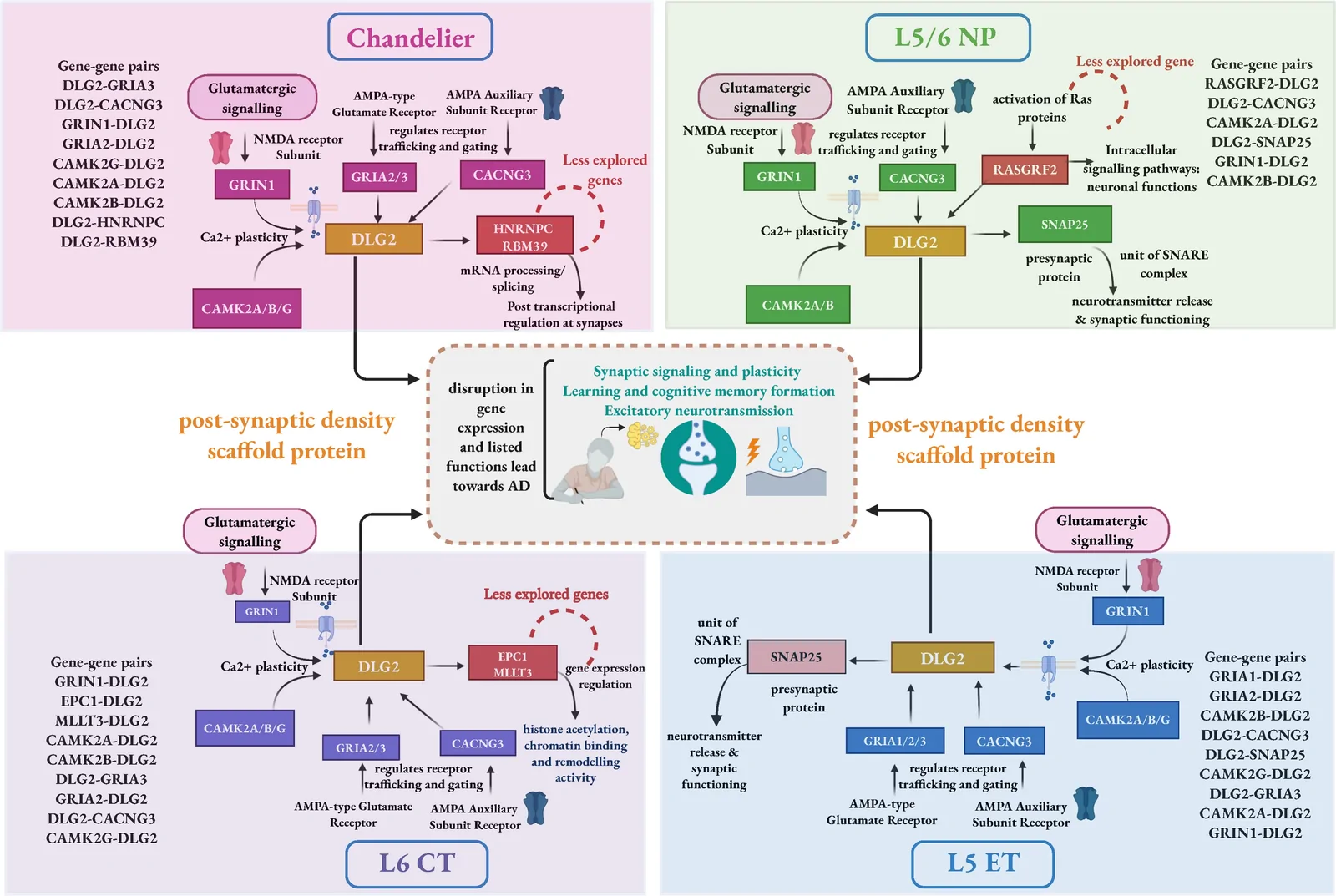
Multi-cell-type hypothesis produced by ADInteractome.

In Chandelier cells, which are GABAergic interneurons that exert strong axo-axonic inhibition on pyramidal neurons [46], DLG2 connects with glutamate receptor subunits (GRIA2, GRIA3, and GRIN1), regulatory subunits (CACNG3), and signaling kinases (CAMK2A, CAMK2B, and CAMK2G). These interactions support receptor trafficking, calcium influx, and synaptic strengthening, all of which are disrupted early in AD. Additionally, DLG2 links to RNA-binding proteins HNRNPC and RBM39, suggesting a potential coupling between synaptic activity and nuclear RNA processing, such as alternative splicing. While RBM39 lacks direct association with AD, its function in splicing may point to emerging mechanisms in neurodegeneration where the relationship between AD and alternative splicing have been observed [47].

In L5/6 NP neurons, DLG2 maintains similar synaptic interactions, engaging GRIN1, CAMK2A/B, CACNG3, and SNAP25. The presence of RASGRF2, a calcium-responsive guanine nucleotide exchange factor whose Rac-GEF activity couples NMDA receptor calcium flux to enhanced synaptic transmission [48], implicates DLG2 in activity-dependent synaptic signaling. SNAP25, a presynaptic SNARE protein required for vesicle fusion and neurotransmitter release, was also co-ranked with DLG2. Because DLG2 is a postsynaptic scaffold, this pairing more likely reflects coordinated pre- and postsynaptic gene programs within the same neuron than a direct protein interaction; both synaptic compartments are frequently impaired in AD [49, 50].

In L6 CT neurons, which project to thalamic targets, DLG2 exhibits broader functional connectivity. In addition to its canonical partners (GRIN1, GRIA2/3, CAMK2A/B/G, and CACNG3), it also interacts with nuclear regulators EPC1, a component of the NuA4/TIP60 histone acetyltransferase complex, and MLLT3, a chromatin-associated transcriptional regulator [38]. Though MLLT3 is best known for its role in leukemia, its interaction here may reflect a synapse-to-nucleus signaling axis, potentially involving chromatin remodeling relevant to AD pathogenesis.

L5 ET neurons, which project to subcortical targets, show the most extensive DLG2-centered interaction network, including GRIA1–3, GRIN1, CAMK2A/B/G, and SNAP25 (see Fig. 3). This broad module likely supports long-range cortical output. Given that layer 5 pyramidal neurons show early circuit and dendritic spine abnormalities in amyloid mouse models [51–53], disruption of this network may contribute to impaired cortical–subcortical communication.

Collectively, these findings suggest that DLG2 is a central regulator of glutamatergic synaptic integrity across diverse cortical neuron types, including both excitatory projection neurons and inhibitory Chandelier interneurons. Based on gene–pair interaction analysis from the SEA-AD dataset, we hypothesize that DLG2-centered molecular networks are critical for maintaining excitatory synaptic stability. Their disruption, through altered expression, mis-localization, or impaired binding, may underlie early, cell-type-specific circuit dysfunction in AD. This hypothesis is consistent with prior evidence that reduced DLG2 expression impairs hippocampal synaptic integration and plasticity in rodents [54], that DLG2 is a risk gene for schizophrenia and related neurodevelopmental disorders [39], and that rare variants in DLG2 have recently been associated with AD [25]. By integrating single-cell transcriptomics with gene interaction mapping, this study outlines a mechanistic framework in which DLG2 coordinates distinct signaling modules across neuronal subtypes. Its dysfunction may serve as a molecular convergence point for the selective vulnerability and synaptic failure characteristic of early AD.

To validate the DLG2-centered hypothesis beyond enrichment analysis and literature support, we additionally cross-referenced DLG2-centered gene pairs against molecular interaction evidence from the Open Targets Platform, including IntAct, Reactome, and STRING. Core synaptic pairs including GRIN1–DLG2, GRIA2–DLG2, GRIA3–DLG2, and CACNG3–DLG2 were supported by curated molecular interaction evidence, whereas several cell-type-specific pairs such as DLG2–SNAP25, RASGRF2–DLG2, and EPC1/MLLT3–DLG2 remain plausible novel candidates. The full evidence is provided in Supplementary Tables S6 and S7.

### Targeted virtual knock-out knock-in experiment confirms functional relevance of DLG2-centered gene pairs

To validate the top-ranked gene–gene interactions identified by ADInteractome in support of the multi-cell-type hypothesis, we perform a ***virtual knock-out knock-in experiment*** focusing on DLG2-centered pairs. The experiment tests whether introducing a gene–gene pair into cells within the snRNA-seq dataset leads to a measurable improvement in the Celluformer model’s accuracy in predicting AD association. A significant increase in predictive performance after knock-out and knock-in suggests that the gene pair may be associated with AD. The intuition behind this procedure is to perform a virtual knock-out of genes in cells, preserving only basic yet potentially AD-irrelevant genes in each cell, to ensure that every cell has some genes expressed. Next, we perform a virtual knock-in to see how the Celluformer model’s performance would be significantly improved when ingesting core gene–gene pairs in the multi-cell-type hypothesis.

To isolate the predictive value of ADInteractome-suggested interactions, we followed the virtual knock-out knock-in protocol described in Methods. We first constructed a base dataset by masking all non-base genes while keeping model weights fixed, which, as expected, reduced accuracy relative to the original full dataset. We then virtually knocked in individual DLG2-partner pairs by restoring their original expression values wherever expressed, leaving all other non-base genes masked. As shown in Table 2, median recovery reached 1.1278 percentage points (pp) in L5_ET neurons and 0.8939 pp in L6_CT neurons, with positive median recovery in L5_6_NP cells (0.5486 pp). The single most beneficial knock-in was DLG2–GRIA3 in L6_CT neurons, which restored accuracy by 3.3520 pp. By contrast, Chandelier interneurons showed no median recovery, although DLG2–GRIA3 still recovered 2.3070 pp in the best case.

**Table 2:** Virtual knock-in accuracy changes (*Δ *Acc, percentage points relative to the masked baseline) for DLG2-centered gene pairs.

Cell type	Gene pair	*Δ * Acc (pp)	Median *Δ *Acc (pp)
Chandelier	DLG2-GRIA3	2.3070	0.0000
	DLG2-CACNG3	0.1775	–
	GRIN1-DLG2	1.2422	–
	GRIA2-DLG2	−0.4437	–
	CAMK2G-DLG2	−0.3549	–
	CAMK2A-DLG2	0.0000	–
	CAMK2B-DLG2	0.0000	–
	DLG2-HNRNPC	−0.2662	–
	DLG2-RBM39	0.8873	–
L5_6_NP	RASGRF2-DLG2	−1.1970	0.5486
	DLG2-CACNG3	0.7481	–
	CAMK2A-DLG2	−0.4489	–
	DLG2-SNAP25	0.8978	–
	GRIN1-DLG2	1.6958	–
	CAMK2B-DLG2	0.3491	–
L5_ET	GRIA1-DLG2	1.1278	1.1278
	GRIA2-DLG2	0.0000	–
	CAMK2B-DLG2	1.1278	–
	DLG2-CACNG3	1.1278	–
	DLG2-SNAP25	1.1278	–
	CAMK2G-DLG2	0.0000	–
	DLG2-GRIA3	3.0075	–
	CAMK2A-DLG2	0.3759	–
	GRIN1-DLG2	1.5038	–
L6_CT	GRIN1-DLG2	2.8492	0.8939
	EPC1-DLG2	1.1173	–
	MLLT3-DLG2	1.1732	–
	CAMK2A-DLG2	−1.2291	–
	CAMK2B-DLG2	0.8380	–
	DLG2-GRIA3	3.3520	–
	GRIA2-DLG2	−2.2905	–
	DLG2-CACNG3	0.8939	–
	CAMK2G-DLG2	0.2235	–

As a control for potential circularity, we compared CelluFormer-selected pairs against randomly sampled gene pairs using the same knock-in procedure (Supplementary Table S2). CelluFormer-selected pairs produced positive mean *Δ *Acc across all four tested cell types, whereas random pairs produced negative mean *Δ *Acc. The difference was significant in L5_ET (*P=.0028*) and L6_CT (*P=.0121*) using a one-sided Mann–Whitney U test, with the same direction of effect in Chandelier and L5_6_NP. This control indicates that performance recovery is not simply a generic consequence of restoring any two genes into the masked input.

## Discussion

This study introduces ADInteractome, an interpretable AI framework that discovers AD-associated gene–gene interactions by analyzing internal interaction states of neural networks trained on snRNA-seq data. ADInteractome is grounded in the hypothesis that if a neural network can accurately classify whether a cell is AD-associated based on its gene expression, then the model’s internal representations must reflect biologically meaningful gene interactions. By extracting and ranking these internal interaction states, ADInteractome reveals cell-type-specific gene circuits associated with AD pathology.

We emphasize that ADInteractome is intended as a hypothesis-generation framework rather than a direct assay of mechanistic molecular binding. In particular, attention-derived neural interaction states should be interpreted as model-based prioritization signals. Attention can be influenced by expression prevalence, sparsity, token competition, scaling, and architectural choices. For this reason, we aggregate interaction states across cells, layers, and heads; normalize Transformer scores by gene-pair co-occurrence; and evaluate the ranked pairs using external biological databases, enrichment analysis, network topology, and virtual perturbation controls.

The classification models trained within ADInteractome, particularly lightweight Transformer architectures, achieved high accuracy across 18 neuronal cell types, with all 18 models surpassing a weighted F1 score of 0.9. This performance supports the foundational premise of the framework: neural networks can capture gene–gene interactions sufficient for robust disease association classification at the cellular level. By analyzing the internal structure of these models, we extracted pairwise interaction scores that reflect latent dependencies among genes. These scores revealed gene–gene pairs that are significantly enriched for known AD-associated interactions compared with traditional co-expression networks.

A key insight from the interaction graphs derived from ADInteractome is the identification of DLG2 as a shared hub gene across multiple cortical neuron types, including Chandelier, L5 ET, L6 CT, and L5/6 NP neurons. DLG2 consistently forms interaction modules with glutamatergic receptor components (GRIA2, GRIA3, and GRIN1), calcium signaling mediators (CAMK2A/B/G), the AMPA receptor auxiliary subunit CACNG3 (TARP *γ *-3), and the presynaptic SNARE protein SNAP25, highlighting its role in maintaining synaptic transmission and plasticity. The absence of DLG2 in L6 IT Car3 neurons points to cell-type-specific regulatory mechanisms that may underlie differential susceptibility to AD.

Importantly, ADInteractome uncovered novel interaction partners of DLG2, including RNA-binding proteins (RBM39 and HNRNPC) and chromatin regulators (EPC1 and MLLT3), suggesting a broader role for DLG2 in coupling synaptic signaling to gene regulatory networks. These findings raise testable hypotheses about how disruptions in DLG2-centered circuits contribute to early synaptic dysfunction in AD. Virtual knock-out/knock-in perturbation analyses further supported the predictive relevance of these circuits, as restoring selected DLG2-associated gene pairs recovered AD-classification accuracy from a masked baseline. The strongest recovery was observed for DLG2–GRIA3, and random gene-pair controls showed that the recovery was not a generic consequence of adding any two genes back into the masked input. These findings highlight the potential of our approach to identify novel drug targets and effective combination therapy strategies by simultaneously targeting both genes within each discovered gene pair.

Our study highlights the potential of AI-based internal state analysis to identify functional, cell-type-specific gene circuits that may be overlooked by conventional methods. By prioritizing interactions learned directly from the data, ADInteractome bypasses reliance on predefined biological priors and enables the discovery of context-specific networks that better reflect disease-specific biology.

We also clarify the intended scope of the hub-based analysis used above. Identifying DLG2 as a recurrent hub is an exploratory and interpretive summary that organizes high-ranking ADInteractome gene pairs into candidate modules for literature-grounded pathway interpretation; it is not a standalone network-inference procedure. We acknowledge that degree-based hubness has limited power to establish directionality or to capture indirect, higher-order, or context-dependent dependencies among genes. More sophisticated topological approaches [55, 56] may complement the current hub summary in future work.

Despite these contributions, several limitations remain. First, model performance depends on the availability of sufficient data per cell type; rare cell populations with few observations remain underrepresented. Second, while ADInteractome successfully ranks known and novel interactions, experimental validation will be necessary to confirm the functional roles of high-ranking gene pairs. In particular, the random gene-pair control shows that the accuracy recovery observed in the virtual knock-in/knock-out analysis is not a generic consequence of restoring arbitrary gene pairs. This control reduces but does not fully eliminate circularity, since both the discovery of candidate interactions and their perturbation-based evaluation rely on the same trained CelluFormer model. The virtual perturbation results should therefore be interpreted as a model-based sanity check rather than as independent biological validation. Finally, this framework has so far been applied to a single dataset. Extending ADInteractome to multi-cohort or multimodal data, such as integrating scATAC-seq or spatial transcriptomics, may further enhance its generalizability and biological resolution. Although stratified analyses across sex and donor subsets (Supplementary Tables S3 and S4) support the robustness of the inferred rankings within SEA-AD, validation on independent AD cohorts remains an important next step. These stratified analyses demonstrate stability of the rankings across selected biological strata, but they are not equivalent to formal covariate-adjusted modeling or region-stratified analysis: they do not fully account for potential confounding by donor, sex, batch, brain region, or neuropathological stage. Future work should incorporate explicit covariate-adjusted models and region-stratified evaluation, ideally across independent cohorts, to more rigorously assess the generalizability of ADInteractome-derived interactions. Future work should also compare ADInteractome with perturbation-based, chromatin-informed, and regulatory-network methods in settings where those additional data modalities are available.

## Conclusion

ADInteractome offers a scalable, interpretable, and data-driven framework for mapping disease-relevant gene–gene interactions in single-cell transcriptomic data. By leveraging internal interaction states of neural networks, it identifies robust molecular circuits underlying AD and highlights DLG2-centered synaptic modules as promising targets for further investigation. This approach provides a foundation for hypothesis generation and experimental prioritization in AD research.

**Key Points**

- ADInteractome discovers Alzheimer’s disease (AD)-associated gene interactions by extracting neural interaction states from deep learning models trained on single-nucleus RNA-seq data.
- The framework reveals cell-type-specific gene circuits supported by existing literature and virtual knock-out/knock-in experiments.
- ADInteractome outperforms traditional co-expression methods in enrichment for known AD-associated interactions and uncovers novel candidate interaction patterns for mechanistic hypothesis generation.

## Supplementary Material

ADInteractome_supplementary

## Data Availability

The SEA-AD snRNA-seq data analyzed in this study are publicly available at the https://sea-ad-single-cell-profiling.s3.amazonaws.com/index.html#MTG/RNAseq/previous_objects/. Detailed descriptions of the model architectures and dataset configurations are provided in the Supplementary Information. The ADInteractome source code, environment specification, and usage instructions are publicly available at https://github.com/Evan810828/ADInteractome.
