# Supplementary material for: ADInteractome: discovering Alzheimer’s disease-associated gene interactions from single-nucleus RNA-seq data through neural interaction states
Source: Brief Bioinform. 2026 Nov 2;27(6):bbag601. doi: 10.1093/bib/bbag601 (PMC13725928; doi:10.1093/bib/bbag601)
Supplement: Supplementary file 1 [file adinteractome_supplementary.pdf]

Supplementary Information

ADInteractome: discovering Alzheimer's disease-associated gene interactions from single-nucleus RNA-seq data through neural interaction states

Yifan Wu[1,*], Neha Maurya[2,*], Yuntao Yang[3], Wenbo Chen[2], Guanchu Wang[4], Zirui Liu[5], Xia Hu[6], W. Jim Zheng[2,†], Zhaozhuo Xu[7,†]

[1]College of Science and Engineering, University of Minnesota Twin Cities, Mayo D528, 420 Delaware St SE, Minneapolis, MN 55455, United States

[2]McWilliams School of Biomedical Informatics, UTHealth Houston, 7000 Fannin St, Suite 600, Houston, TX 77030, United States

[3]MD Anderson Cancer Center, 1515 Holcombe Blvd, Houston, TX 77030, United States

[4]Department of Computer Science, University of North Carolina at Charlotte, Woodward Hall, 9201 University City Blvd, Charlotte, NC 28223, United States

[5]Department of Computer Science & Engineering, University of Minnesota Twin Cities, 4-192 Keller Hall, 200 Union Street SE, Minneapolis, MN 55455, United States

[6]Department of Computer Science, Rice University, Duncan Hall 3122, 6100 Main Street, Houston, TX 77005, United States

[7]School of Computing, Stevens Institute of Technology, Gateway Center North, 1 Castle Point Terrace, Hoboken, NJ 07030, United States

[*]Yifan Wu and Neha Maurya contributed equally to this work.

[†]Corresponding authors. W. Jim Zheng, E-mail: Wenjin.J.Zheng@uth.tmc.edu; Zhaozhuo Xu, E-mail: zxu79@stevens.edu

Figure numbers in this document (Figures 1–3) refer to the figures of the main text.

S1 Experiment Details

Transformer Configurations: In this work, we used the standard multi-head self-attention introduced in [1]. Moreover, we perform padding on each batch of training and inference of single-cell data. Accordingly, we introduce a padding mask in the attention mechanism to avoid computation on the padded position. We represent each input sequence by employing a lookup table that transforms a comprehensive vocabulary of 36,601 genes into 128-dimensional embedding vectors. These vectors are subsequently processed through a series of 4 Transformer encoder blocks. Each encoder block is equipped with 8 attention heads, a 512-dimensional feedforward layer, and a dropout layer in a ratio of 0.1. The processed outputs are then directed to a linear classification layer, which is tasked with predicting labels indicative of Alzheimer's Disease conditions. We adopted the Adam Optimization Algorithm to minimize the loss function [2]. The model is trained under a learning rate of 1e-5, and the batch size of our data loader is set as 128.

MLP Configurations: The MLP consists of 2 hidden layers, with 128 and 256 hidden units, respectively. Each hidden layer is followed by a dropout and a Softplus module. The MLP is trained under a learning rate of 1e-4 and the batch size of our data loader is set as 128. We adopted the Adam Optimization Algorithm to minimize the loss function [2].

Diamond Baseline Configurations: We evaluate a Diamond-inspired [3] baseline on curated single-cell AnnData files from our gene–gene interaction benchmark. Expression matrices are densified where needed and truncated to a fixed gene set of size $g = 50$ (the most important 50 features calculated from Transformers' self-attention score). Unless otherwise stated, a mini-batch size of 128 and GPU acceleration are used. Following the Diamond formulation, the method augments

each input with a knockoff copy to form $(X, \tilde{X})$, enabling contrastive modeling of original-versus-knockoff contributions. For efficiency, we adopt a deterministic "self-knockoff" where $\tilde{X} = X$, resulting in $2g$-dimensional inputs per cell. This proxy preserves symmetric contrast but does not implement the generative or FDR-controlled components of full Diamond; it serves purely as a ranked-score baseline.

The classifier follows a DeepPINK-style [4] MLP backbone over the $2g$-dimensional inputs with two hidden layers of 128 and 64 units. Training uses the Adam optimizer [2] (learning rate 10^{-3}), batch size 128, and a maximum of 20 epochs under PyTorch Lightning defaults (no early stopping or scheduler). Although labels are binary, we employ mean-squared-error loss as a simple surrogate; accuracy is monitored via argmax over logits. Random seeds are fixed for reproducibility. After training, we compute global feature attributions and pairwise gene–gene interaction scores for the first g genes using the model's interaction routine. Per-sample interaction magnitudes are averaged across the evaluation set, and index sets for original–knockoff and knockoff–knockoff pairs are recorded to support calibration and diagnostics.

Pearson and CS-CORE Baseline Configurations: For Pearson correlation, we computed gene–gene correlation scores using `numpy.corrcoef`. Because the expression matrix is organized as cells $\times$ genes, genes were treated as variables across cells when computing the correlation matrix. The resulting gene–gene correlation scores were converted into ranked gene-pair lists for the same enrichment analysis pipeline.

For CS-CORE, we used the official Python implementation with default settings. Specifically, we used `CSCORE(adata, gene_index, seq_depth=None)`; under this default configuration, sequencing depth is computed from `adata.raw.X.sum(axis=1)` when `seq_depth` is not provided, and the implementation calls `CSCORE_IRLS(..., post_process=True)` internally. The returned co-expression estimate matrix was used to rank gene–gene pairs.

Gene Enrichment Analysis Configurations: We conducted gene set enrichment analysis (GSEA) using the GSEApy [5] package (v1.1.3) with parameters *min_size* = 1, *max_size* = 1e9, *permutation_num* = 1000, *seed* = 6, and *outdir* = None. Custom gene sets were constructed from BioGRID [6] and DisGeNET [7] reference files in CSV format. To maintain experimental reproducibility, all stochastic operations were seeded, and nominal p-values were clipped at a lower bound of 10^{-3}. The resulting metrics include enrichment score (ES), normalized enrichment score (NES) [8], nominal p-value, tag percentage, and gene percentage.

S1.1 Sensitivity and control analyses

Temperature-sensitivity analysis: To evaluate whether the Transformer-derived interaction ranking depends on a single narrowly tuned temperature value, we recomputed the interaction scores and BioGRID enrichment analysis across six values of T in L5 ET and L6 CT cells. $T = 3.0$ yielded the largest NES in both cell types and was therefore used as the default setting.

Random gene-pair knock-in control: To test whether performance recovery is specific to CelluFormer-selected pairs rather than a generic effect of restoring any genes, randomly sampled gene pairs were evaluated with the same virtual knock-in protocol. A one-sided Mann–Whitney U test compared whether CelluFormer-selected pairs produced larger ΔAcc than random pairs.

Stratified robustness analysis: We recomputed CelluFormer attention-derived gene-pair rankings separately within biological strata and compared each stratum-specific ranking with the full-data ranking using top-K Jaccard overlap. Sex-stratified subsets were evaluated for L5_ET and L6_CT, and donor-stratified subsets were evaluated for the three largest L6_CT donors. We performed this robustness analysis under temperature $T = 1.0$.

Table S1: Temperature-sensitivity ablation of the attention-derived interaction score.

Cell type	Temperature	NES	p-value
L5 ET	$T = 0.25$	0.9734	0.547
L5 ET	$T = 0.5$	0.6964	0.987
L5 ET	$T = 1.0$	1.0566	0.342
L5 ET	$T = 2.0$	1.0131	0.449
L5 ET	$T = 3.0$	1.4322	0.358
L5 ET	$T = 4.0$	1.0590	0.330
L6 CT	$T = 0.25$	0.8236	0.876
L6 CT	$T = 0.5$	0.8108	0.893
L6 CT	$T = 1.0$	0.8834	0.779
L6 CT	$T = 2.0$	0.9659	0.608
L6 CT	$T = 3.0$	1.7292	0.173
L6 CT	$T = 4.0$	1.1428	0.158

Table S2: Random gene-pair knock-in control. Random and CelluFormer columns report mean ΔAcc relative to the masked baseline.

Cell type	Baseline acc	Random pairs mean ΔAcc	CelluFormer mean ΔAcc	MWU p
Chandelier	66.37%	−0.74%	+0.39%	0.0975
L5_ET	70.30%	−0.66%	+1.04%	0.0028
L5_6_NP	48.58%	−0.29%	+0.34%	0.1049
L6_CT	44.64%	−0.72%	+0.77%	0.0121

S2 Additional analysis on the discovered gene interactions

Overview. This supplementary section provides expanded analyses supporting the ADInteractome-derived gene interaction results (Supplementary Tables S5–S7). We summarize pathway enrichment of top-ranked interaction modules, hub-gene and interactor analyses, threshold sensitivity for network construction, and independent database cross-validation of the DLG2-centered hypothesis.

S2.1 Gene ontology and pathway analysis of top-ranked gene-gene interactions in snRNA-Seq SEA-AD dataset

k-means clustering was performed in STRING on the protein-protein interaction networks built from the unique genes in the top 1000 ranked gene-gene pairs of each cell type in the SEA-AD snRNA-Seq dataset[9]. The number of clusters was set to $k = 3$ for all five cell types to keep the clustering consistent and comparable. For each cell type, the largest of the three clusters (the one containing the most genes) was selected for downstream analysis. The number of unique genes contributing to these networks for each cell type were as follows: Chandelier (756), L6 CT (775), L6 IT Car3 (843), L5 ET (854), and L5/6 NP (501). From these, the top gene clusters identified through clustering consisted of 535 genes for Chandelier, 580 for L6 CT, 643 for L6 IT Car3, 659 for L5 ET, and 327 for L5/6 NP.

Gene set enrichment analysis using the DISEASES database for disease-gene association revealed distinct enrichment patterns for each cell type[9, 10]. In Chandelier cells, the top gene cluster was significantly enriched for neurological conditions such as epilepsy, brain disease, neurodegenerative disease, brain system disease, and nervous system disease. L6 CT cells also showed enrichment for several overlapping categories, including brain disease, neurodegenerative disease, brain system disease, and nervous system disease. For L6 IT Car3 cells, the enrichment profile was broader, showing associations with autosomal genetic disorders, peripheral nervous system disease, autosomal dominant disease, epilepsy, brain disease, mental health disorders, neurodegenerative

Table S3: Stratified robustness of CelluFormer-derived gene-pair rankings. Values report mean top-K Jaccard overlap between stratum-specific rankings and the full-data ranking.

Cell type	Axis	Mean J@500	Mean J@1000	Mean J@5000
L5_ET	Sex	0.757	0.762	0.725
L6_CT	Sex	0.761	0.752	0.734
L6_CT	Donor	0.516	0.518	0.489

Table S4: Per-stratum BioGRID enrichment analysis. NES and p-values were computed for the full-data baseline and each stratum-specific ranking.

Cell type	Axis	Stratum	NES	p-value
L5_ET	Baseline	FULL_T1.0	1.0566	0.342
L5_ET	Sex	Female	1.0346	0.392
L5_ET	Sex	Male	1.0387	0.398
L6_CT	Baseline	FULL_T1.0	0.8834	0.779
L6_CT	Sex	Female	0.9284	0.674
L6_CT	Sex	Male	0.9870	0.529
L6_CT	Donor	H20_33_017	0.9254	0.703
L6_CT	Donor	H20_33_018	0.8933	0.794
L6_CT	Donor	H20_33_039	1.0339	0.413

disease, nervous system disease, and central nervous system disease. Similarly, L5 ET cells exhibited enrichment for a wide range of conditions, including autosomal and chromosomal diseases, genetic disorders, peripheral nervous system disease, autosomal dominant disease, brain disease, epilepsy, mental health disorders, neurodegenerative disease, nervous system disease, and central nervous system disease. The L5/6 NP cells have shown disease associations predominantly involving mental health disorders, brain disease, nervous system disease, and central nervous system disease. These cell-type–specific enrichments highlight the potential involvement in Alzheimer's disease pathology.

Reactome pathway enrichment analysis of the top gene clusters from each SEA-AD cell type revealed a highly conserved set of pathways associated with neuronal function, synaptic transmission, and plasticity. Across all five cell types-Chandelier, L6_CT, L6 IT Car3, L5 ET, and L5/6 NP—the most significantly enriched pathways included neurotransmitter receptor activity and postsynaptic signal transmission, transmission across chemical synapses, trafficking of AMPA receptors, neuronal system signaling, activation and post-activation events of NMDA receptors, glutamate binding and receptor activation, long-term potentiation, and trafficking of GluR2-containing AMPA receptors. These pathways are fundamental to glutamatergic synaptic function and plasticity, highlighting conserved molecular programs across both excitatory and inhibitory cortical neuron types relevant to AD pathology.

Despite this common core of synaptic signaling pathways, some cell types exhibited additional or context-specific enrichments. For instance, Chandelier cells were uniquely enriched for PLC-β mediated events and muscle contraction, likely reflecting shared calcium-signaling and ion-channel genes rather than contractile processes per se. L6 IT Car3 cells also showed enrichment in pathways related to regulation of insulin secretion, integration of energy metabolism, and L1CAM interactions, implicating metabolic and adhesion-related signaling mechanisms. Additionally, L5/6 NP cells were enriched for Ras activation upon Ca^{2+} influx through NMDA receptors and interactions between L1 and ankyrins, pointing to specific calcium-mediated and cytoskeletal signaling pathways.

S2.2 Identification of key gene–gene interactions through hub gene and interactors analysis

To identify critical regulatory components within each cell type, we performed hub gene analysis using the Maximal clique centrality (MCC) algorithm on the top-ranking gene clusters derived from k-means clustering across all SEA-AD cell types [11]. For each cell type, the top 10 hub genes were identified based on their centrality and connectivity within the gene interaction network shown in Figure 2.

In Chandelier cells, the prominent hub genes included CAMK2A, CAMK2G, CACNG3, GRIA3, GRIA2, DLG2, CAMK2B, GRIN1, HNRNPC, and RBM39, representing a strong enrichment for calcium/calmodulin-dependent signaling and glutamatergic synapse components. The L6 CT cell type shared several of these genes, including GRIN1, CAMK2G, CACNG3, DLG2, CAMK2B, GRIA3, and GRIA2, with additional hub genes such as EPC1 and MLLT3, which may suggest cell-specific chromatin regulatory mechanisms. In L6 IT Car3 cells displayed a distinct set of RNA-binding and splicing-related hub genes, including RBM25, TRA2B, SRSF10, HNRNPC, RPS2, RBM39, HNRNPU, SFPQ, SRSF1, and SREK1, indicating a strong influence of post-transcriptional regulation in this subtype. The L5 ET cell type exhibited a hybrid profile with synaptic and signaling-related hub genes, including GRIN1, DLG2, GRIA3, CAMK2A, CAMK2B, CAMK2G, SNAP25, CACNG3, GRIA1, and GRIA2, suggesting prominent excitatory signaling involvement. Finally, L5/6 NP cells showed a closely related hub gene profile to L5 ET, encompassing GRIA2, GRIA3, GRIN1, SNAP25, DLG2, CAMK2G, CAMK2A, RASGRF2, CAMK2B, and CACNG3. The consistent presence of CAMK2 family members, ionotropic glutamate receptors, and synaptic scaffolding proteins across multiple cell types underscores their potential as central molecular players in AD-associated neuronal dysfunction.

The hub genes were further evaluated for their known interactors, focusing on shared biological pathways to establish functional relevance [12].The gene interaction analysis has revealed different interactors for each cell type for the SEA-AD dataset which are often involved in the similar type of functions and biological pathways, shown in Figure 2.

To evaluate the relevance of hub gene interactions, the gene–gene pairs involving identified hub genes were mapped back to the top 1000 ranked gene–gene pairs for each cell type based on ranking scores. The resulting overlap is illustrated in Figure 2. Among all cell types, DLG2 consistently emerged as a central interactor, displaying the highest number of gene–gene associations across multiple cell types. Notably, the L6 IT Car3 cell type did not include DLG2 among its identified hub genes and, correspondingly, showed no gene-gene pair interactions within its top 1000 ranked gene-gene pairs. This exception highlights the cell type-specific nature of gene interaction networks and underscores the prominent role of DLG2 in AD. These findings suggest that DLG2 may serve as a core scaffold or regulatory node in cell-type-specific signaling processes relevant to AD pathology.

S2.3 Threshold sensitivity of network construction

The primary interaction graphs were constructed from the top 1,000 ranked gene–gene pairs because this threshold preserved enough edges for clustering and hub detection while maintaining interpretable sparse modules. To evaluate sensitivity to this choice, we repeated hub-gene mapping at top 800 and top 1,500 cutoffs across the five cortical cell types.

At the top 800 cutoff, the DLG2-centered architecture was preserved across the four DLG2-positive cell types (Chandelier, L6 CT, L5 ET, and L5/6 NP), indicating that the primary biological signal does not depend on exactly 1,000 edges. At the top 1,500 cutoff, the additional lower-ranked edges introduced more diffuse ribosomal and RNA-processing modules in several cell types,

Table S5: Hub gene mapping and gene–gene pair identification across three interaction thresholds.

Cell type	Top 800	Top 1,000 (primary)	Top 1,500	Key observation
Chandelier	DLG2 hub preserved; all core pairs replicated	DLG2 hub; 9 AD-relevant pairs	DLG2 hub partially preserved; GRIN2D and CACNG2 emerge	Stable at 800 and 1,000; partial dilution at 1,500
L6 CT	DLG2 hub; SNAP25/KMT2A replace EPC1/MLLT3 at periphery	DLG2 hub; EPC1 and MLLT3 pairs; 9 AD-relevant pairs	Ribosomal cluster; no DLG2 pairs found	AD-relevant hub topology is lost at 1,500
L6 IT Car3	No DLG2 pairs	No DLG2 pairs	No DLG2 pairs	Threshold-independent negative subtype
L5 ET	DLG2 hub; KMT2A replaces GRIA1 at periphery	DLG2 hub; 9 core pairs	Ribosomal cluster; no DLG2 pairs found	AD-relevant hub topology is lost at 1,500
L5/6 NP	DLG2 hub; KMT2A/KMT2C additionally appear	DLG2 hub; 6 core pairs	RNA-processing cluster; no DLG2 pairs found	AD-relevant hub topology is lost at 1,500

diluting the DLG2-centered topology. Edges in the visualization represent GeneMANIA-supported relationships. Physical interactions were weighted by database confidence scores, and additional evidence categories such as co-expression, co-localization, genetic interactions, shared domains, and pathway evidence were retained for biological interpretation. No explicit tie-handling was required at the top 1,000 boundary because adjacent ranked scores differed at sufficient numerical precision.

S2.4 Multi-cell-type hypothesis generation with ADInteractome

AD is characterized by progressive synaptic dysfunction and cognitive decline, with emerging evidence suggesting that distinct neuronal populations may exhibit differential vulnerability to early pathophysiological events [13, 14, 15]. DLG2 (Discs Large MAGUK Scaffold Protein 2), a postsynaptic density protein and member of the MAGUK family, is pivotal for organizing glutamatergic synapses. It functions as a scaffold for AMPA and NMDA receptor complexes and modulates intracellular signaling cascades involved in synaptic plasticity [16, 17]. Our gene interaction analyses reveal that DLG2 forms a core hub across four cortical cell types: Chandelier cells, L5/6 NP (near-projecting), L6 CT (Corticothalamic), and L5 ET (Extratelencephalic) neurons, interacting with a set of conserved partners implicated in neurotransmission, calcium signaling, and synaptic remodeling.

In Chandelier cells, which are GABAergic interneurons that provide powerful axo-axonic inhibition to pyramidal cells [18], DLG2 interacts with glutamate receptor subunits (GRIA2, GRIA3, GRIN1), as well as regulatory subunits such as CACNG3 and signaling kinases CAMK2A, CAMK2B, and CAMK2G. These genes are essential for receptor trafficking, calcium influx, and synaptic strengthening—processes that are dysregulated early in AD. Notably, DLG2 also interacts with RNA-binding proteins HNRNPC and RBM39, suggesting a potential coupling between synaptic activity and nuclear RNA processing, such as alternative splicing, which has been increasingly implicated in neurodegeneration. Although RBM39 has no established association with AD, its role in alternative splicing is consistent with reported splicing dysregulation in AD [19]. In L5/6 NP neurons, DLG2 maintains its core synaptic role by partnering with GRIN1, CAMK2A/B, CACNG3, and SNAP25. The inclusion of RASGRF2, a calcium-responsive guanine nucleotide exchange factor whose Rac-GEF activity couples NMDA receptor calcium flux to enhanced synaptic transmission [20], points toward a role for DLG2 in activity-dependent synaptic signaling. SNAP25, a presynaptic SNARE protein required for vesicle fusion and neurotransmitter release, was also co-ranked with DLG2; because DLG2 is a postsynaptic scaffold, this pairing more likely reflects coordinated

Table S6: Molecular interaction evidence for DLG2-centered gene pairs. Database-supported pairs are separated from candidate interactions with limited direct database evidence.

Gene pair	Cell types	Score	Sources	Validation status
Core synaptic pairs with curated database evidence				
GRIN1–DLG2	Chandelier, L6 CT, L5 ET, L5/6 NP	0.44	IntAct, STRING	Database-supported association with the NMDA receptor complex
GRIA2–DLG2	Chandelier, L6 CT, L5 ET	0.44	IntAct, Reactome, STRING	Database-supported association within the postsynaptic AMPA receptor complex
GRIA3–DLG2	Chandelier, L6 CT, L5 ET	0.44	IntAct, STRING	Database-supported AMPA receptor subunit association
CACNG3–DLG2	Chandelier, L6 CT, L5 ET, L5/6 NP	0.44	IntAct, STRING	Confirmed AMPA auxiliary subunit relationship with DLG2-associated receptor trafficking
Cell-type-specific candidate pairs with limited direct database evidence				
DLG2–SNAP25	L5 ET, L5/6 NP	–	Not detected	Candidate trans-synaptic link involving a presynaptic SNARE protein
EPC1/MLLT3–DLG2	L6 CT only	–	Not detected	Candidate chromatin-remodelling and histone-acetylation module unique to L6 CT
RASGRF2–DLG2	L5/6 NP only	–	Not detected	Candidate Ras GEF and intracellular signaling interaction unique to L5/6 NP
DLG2–HNRNPC/RBM39	Chandelier only	–	Not detected	Candidate RNA splicing or processing module linked to synaptic regulation

Table S7: Reactome pathway evidence for CAMK2A/B/G–DLG2 interactions.

Gene pair	Cell types	Database	Pathway / evidence	FDR
CAMK2A–DLG2	Chandelier, L6 CT, L5 ET, L5/6 NP	Reactome	Long-term potentiation at the postsynaptic density	<0.0027
CAMK2B–DLG2	Chandelier, L6 CT, L5 ET, L5/6 NP	Reactome	Long-term potentiation at the postsynaptic density	<0.0027
CAMK2G–DLG2	Chandelier, L6 CT, L5 ET	Reactome	Long-term potentiation at the postsynaptic density	<0.0027

pre- and postsynaptic gene programs within the same neuron than a direct protein interaction, and both compartments contribute to synaptic transmission deficits observed in AD [21, 22]. In L6 CT neurons, which relay cortical signals to thalamic targets, DLG2 exhibits broader functional versatility. In addition to core glutamatergic components (GRIN1, GRIA2/3, CAMK2A/B/G, CACNG3), it also connects with nuclear regulatory proteins EPC1, a component of the NuA4/TIP60 histone acetyltransferase complex, and MLLT3, a chromatin-associated transcriptional regulator [23], suggesting potential nuclear-synaptic communication or chromatin remodeling roles. While MLLT3 is primarily known for its role in leukemia and hematopoiesis, there is no direct and established link between it and Alzheimer's disease. These interactions may represent an epigenetic layer of synaptic plasticity regulation disrupted in AD. Finally, L5 ET neurons, which project to subcortical targets, show the most extensive DLG2-based network, including AMPA receptor subunits (GRIA1, GRIA2, GRIA3), GRIN1, CAMK2 (A/B/G) isoforms, and SNAP25 as shown in Figure 3. The preservation of this extended DLG2 module underscores its critical role in sustaining long-range cortical output. Given that layer 5 pyramidal neurons show early circuit and dendritic spine abnormalities in amyloid mouse models [24, 25, 26], disruption of this network may impair cortical-subcortical connectivity.

Taken together, the convergent pattern of DLG2 interactions across diverse cortical neuronal

subtypes (Chandelier, L5/6 NP, L6 CT, and L5 ET) suggests that DLG2 acts as a central molecular regulator of synaptic function. On the basis of gene-gene pair interaction analysis from the SEA-AD dataset, we hypothesize that DLG2-centered molecular networks are critical for maintaining excitatory synaptic integrity, and that disruption of these interactions—via altered expression, mislocalization, or impaired protein-protein binding—drives synaptic destabilization, calcium signaling imbalances, and impaired plasticity, ultimately contributing towards the early, cell-type–specific circuit breakdown observed in AD as shown in Figure 3.

This hypothesis is consistent with prior evidence that reduced DLG2 expression impairs hippocampal synaptic integration and plasticity in rodents [27], that DLG2 is a risk gene for schizophrenia and related neurodevelopmental disorders [17], and that rare variants in DLG2 have recently been associated with AD [16]. By integrating single-cell transcriptomic data with gene interaction mapping, this study provides a mechanistic framework in which a common synaptic scaffold—DLG2—coordinates functionally distinct signaling modules across neuron types. Its dysfunction may thus serve as a molecular convergence point for the selective neuronal vulnerability and synaptic failure that hallmark early AD progression.

S2.5 Independent database evidence for DLG2-centered pairs

To provide validation independent of enrichment analysis and narrative literature support, we cross-referenced DLG2-centered gene pairs from the primary analysis against molecular interaction evidence from the Open Targets Platform source databases, including IntAct, Reactome, and STRING.

References

[1] Ashish Vaswani, Noam Shazeer, Niki Parmar, Jakob Uszkoreit, Llion Jones, Aidan N Gomez, Łukasz Kaiser, and Illia Polosukhin. Attention is all you need. *Advances in neural information processing systems*, 30:5998–6008, 2017.

[2] Diederik P. Kingma and Jimmy Ba. Adam: A method for stochastic optimization. In Yoshua Bengio and Yann LeCun, editors, *3rd International Conference on Learning Representations, ICLR 2015, San Diego, CA, USA, May 7-9, 2015, Conference Track Proceedings*, 2015.

[3] Winston Chen, Yifan Jiang, William Stafford Noble, and Yang Young Lu. Error-controlled non-additive interaction discovery in machine learning models. *Nature Machine Intelligence*, 7(9):1541–1554, 2025.

[4] Yang Young Lu, Yingying Fan, Jinchi Lv, and William Stafford Noble. DeepPINK: Reproducible feature selection in deep neural networks. In *Advances in Neural Information Processing Systems*, volume 31. Curran Associates, Inc., 2018.

[5] Zhuoqing Fang, Xinyuan Liu, and Gary Peltz. GSEApy: a comprehensive package for performing gene set enrichment analysis in Python. *Bioinformatics*, 39(1):btac757, 2023.

[6] Rose Oughtred, Chris Stark, Bobby-Joe Breitkreutz, Jennifer Rust, Lorrie Boucher, Christie Chang, Nadine Kolas, et al. The BioGRID interaction database: 2019 update. *Nucleic Acids Research*, 47(D1):D529–D541, 2019.

[7] Janet Piñero, Àlex Bravo, Núria Queralt-Rosinach, Alba Gutiérrez-Sacristán, Jordi Deu-Pons, Emilio Centeno, Javier García-García, Ferran Sanz, and Laura I Furlong. DisGeNET: a comprehensive platform integrating information on human disease-associated genes and variants. *Nucleic Acids Research*, 45(D1):D833–D839, 2016.

[8] Aravind Subramanian, Pablo Tamayo, Vamsi K. Mootha, Sayan Mukherjee, Benjamin L. Ebert, Michael A. Gillette, Amanda Paulovich, Scott L. Pomeroy, Todd R. Golub, Eric S. Lander, and Jill P. Mesirov. Gene set enrichment analysis: a knowledge-based approach for interpreting genome-wide expression profiles. *Proceedings of the National Academy of Sciences of the United States of America*, 102(43):15545–15550, Oct 2005. Epub 2005 Sep 30.

[9] Damian Szklarczyk et al. The STRING database in 2025: protein networks with directionality of regulation. *Nucleic Acids Research*, 53(D1):D730–D737, 2025.

[10] Sune Pletscher-Frankild, Albert Pallejà, Kalliopi Tsafou, Janos X. Binder, and Lars Juhl Jensen. DISEASES: Text mining and data integration of disease–gene associations. *Methods*, 74:83–89, 2015.

[11] Chia-Hao Chin et al. cytoHubba: identifying hub objects and sub-networks from complex interactome. *BMC Systems Biology*, 8(Suppl 4):S11, 2014.

[12] Max Franz et al. GeneMANIA update 2018. *Nucleic Acids Research*, 46(W1):W60–W64, 2018.

[13] Pushpa Tryphena Kamatham, Rashi Shukla, Dharmendra Kumar Khatri, and Lalitkumar K Vora. Pathogenesis, diagnostics, and therapeutics for Alzheimer's disease: Breaking the memory barrier. *Ageing research reviews*, 101:102481, 2024.

[14] Raquel N. Taddei and Karen E. Duff. Synapse vulnerability and resilience underlying Alzheimer's disease. *EBioMedicine*, 112:105557, 2025.

[15] Jifa Zhang et al. Recent advances in Alzheimer's disease: Mechanisms, clinical trials and new drug development strategies. *Signal Transduction and Targeted Therapy*, 9(1):211, 2024.

[16] Dmitry Prokopenko et al. Region-based analysis of rare genomic variants in whole-genome sequencing datasets reveal two novel Alzheimer's disease-associated genes: DTNB and DLG2. *Molecular Psychiatry*, 27(4):1963–1969, 2022.

[17] Bret Sanders et al. Transcriptional programs regulating neuronal differentiation are disrupted in DLG2 knockout human embryonic stem cells and enriched for schizophrenia and related disorders risk variants. *Nature Communications*, 13(1):27, 2022.

[18] Benjamin Compans and Juan Burrone. Chandelier cells shine a light on the formation of GABAergic synapses. *Current Opinion in Neurobiology*, 80:102697, 2023.

[19] Towfique Raj, Yang I. Li, Garrett Wong, Jack Humphrey, Minghui Wang, Satesh Ramdhani, Ying-Chih Wang, Bernard Ng, Ishaan Gupta, Vahram Haroutunian, Eric E. Schadt, Tracy Young-Pearse, Sara Mostafavi, Bin Zhang, Pamela Sklar, David A. Bennett, and Philip L. De Jager. Integrative transcriptome analyses of the aging brain implicate altered splicing in Alzheimer's disease susceptibility. *Nature Genetics*, 50(11):1584–1592, Nov 2018.

[20] Brandon Schwechter, Christian Rosenmund, and Kimberley F. Tolias. RasGRF2 Rac-GEF activity couples NMDA receptor calcium flux to enhanced synaptic transmission. *Proceedings of the National Academy of Sciences*, 110(35):14462–14467, 2013.

[21] Heather Wood. SNAP25—an early biomarker in AD and CJD. *Nature Reviews Neurology*, 18(10):575, 2022.

[22] Hamid Ceylan. Integrated bioinformatics analysis to identify alternative therapeutic targets for Alzheimer's disease: insights from a synaptic machinery perspective. *Journal of Molecular Neuroscience*, 72(2):273–286, 2022.

[23] Marilyn Safran, Irina Dalah, Justin Alexander, Naomi Rosen, Tsippi Iny Stein, Michael Shmoish, Noam Nativ, Iris Bahir, Tirza Doniger, Hagit Krug, et al. GeneCards Version 3: the human gene integrator. *Database*, 2010:baq020, 2010.

[24] Chang Chen et al. Disrupted maturation of prefrontal layer 5 neuronal circuits in an Alzheimer's mouse model of amyloid deposition. *Neuroscience Bulletin*, 39(6):881–892, 2023.

[25] Mario M. Dorostkar et al. Analyzing dendritic spine pathology in Alzheimer's disease: problems and opportunities. *Acta Neuropathologica*, 130:1–19, 2015.

[26] Chang Chen et al. Early impairment of cortical circuit plasticity and connectivity in the 5XFAD Alzheimer's disease mouse model. *Translational Psychiatry*, 12(1):371, 2022.

[27] Simonas Griesius et al. Reduced expression of the psychiatric risk gene DLG2 (PSD93) impairs hippocampal synaptic integration and plasticity. *Neuropsychopharmacology*, 47(7):1367–1378, 2022.
